# A first insight into the genetics of maturity trait in Runner × Virginia types peanut background

**DOI:** 10.1038/s41598-022-19653-z

**Published:** 2022-09-10

**Authors:** Srinivas Kunta, Pragna Parimi, Yael Levy, Chandrasekhar Kottakota, Ilan Chedvat, Ye Chu, Peggy Ozias-Akins, Ran Hovav

**Affiliations:** 1 Department of Vegetable and Field Crops, Institute of Plant Sciences, Agriculture Research Organization-The Volcani Center, HaMaccabim Road, POB 15159, 7505101 Rishon LeZiyyon, Israel; 2 The Robert H. Smith Faculty of Agriculture, Food and Environment, The Hebrew University of Jerusalem, POB 12, 7610001 Rehovot, Israel; 3 Department of Horticulture and Institute of Plant Breeding, Genetics and Genomics, University of Georgia, Tifton, GA 31793 USA

**Keywords:** Genetic linkage study, Plant genetics

## Abstract

'Runner' and 'Virginia', the two main market types of *Arachis hypogaea* subspecies *hypogaea*, differ in several agricultural and industrial characteristics. One such trait is time to maturation (TTM), contributing to the specific environmental adaptability of each subspecies. However, little is known regarding TTM's genetic and molecular control in peanut in general, and particularly in the Runner/Virginia background. Here, a recombinant inbred line population, originating from a cross between an early-maturing Virginia and a late-maturing Runner type, was used to detect quantitative trait loci (QTL) for maturity. An *Arachis* SNP-array was used for genotyping, and a genetic map with 1425 SNP loci spanning 24 linkage groups was constructed. Six significant QTLs were identified for the maturity index (MI) trait on chromosomes A04, A08, B02 and B04. Two sets of stable QTLs in the same loci were identified, namely *qMIA04a,b and qMIA08_2a,b* with 11.5%, 8.1% and 7.3%, 8.2% of phenotypic variation explained respectively in two environments. Interestingly, one consistent QTL, *qMIA04a,b*, overlapped with the previously reported QTL in a Virginia × Virginia population having the same early-maturing parent ('Harari') in common. The information and materials generated here can promote informed targeting of peanut idiotypes by indirect marker-assisted selection.

## Introduction

Peanut (*Arachis hypogaea* L.) is an economically important grain legume widely cultivated in tropical, subtropical, and warm temperate regions. It is a global nutritional source of food and edible oil and serves as a good fodder source. Domesticated peanut is a self-pollinated allotetraploid (AABB genome, 2n = 4x = 40) that evolved from a natural hybridization event between two diploid progenitors, *A. duranensis* (AA, 2n = 2x = 20) and *A. ipaensis* (BB, 2n = 2x = 20)^1,2^. The commercial *A. hypogaea* cultivars are categorized into four market types, in which the 'Spanish' and 'Valencia' types belong to the subspecies *fastigiata*, while the 'Virginia' and 'Runner' market-types belong to the subspecies *hypogaea*. The two subspecies differ by plant architecture, flowering pattern and seed size^3^. *A. hypogaea* ssp. *hypogaea* types exhibit an alternative flowering pattern and indeterminate spreading or bunch habit and relatively big seeds, whereas *A. hypogaea* ssp. *fastigiata* are characterized by a sequential flowering pattern, erect growth habit and small seeds^4^.

The growing period, or time to maturation (TTM), is another essential trait for crop adaptability and yield in peanut. While the environmental conditions and agricultural practices influence TTM^5,6^, it has a significant genetic component, as evident by the wide range of TTM among the different market types^7^. In general, ssp. *hypogaea* types are late in fruit maturation [130–170 days post-planting (DPP)], whereas ssp. *fastigiata* are characterized by early fruit maturation (90–120 DPP)^4^. Variation in TTM also exists between Virginia and Runner, the two *A. hypogaea* ssp*. hypogaea* market types. The Runner type has become the dominant peanut type grown in the United States due to the introduction of varieties (e.g., Florunner) in the early 1970s with a substantial increase in peanut yields. The name "Runner" originated from these cultivars' spreading growth habit. Runners have gained wide acceptance because of a desirable range in kernel size, particularly for the peanut butter industry^8^. This market type is grown mainly in US states such as Georgia, Alabama, Florida, Arkansas, Mississippi, Oklahoma and Texas. It accounts for over 80% of total USA production. The Virginia-type peanuts have a larger kernel size than the Runner types. They account for most of the "in-shell" peanut market. When shelled, the large kernels are sold as salted peanuts. Virginia peanuts are grown mainly in southeastern Virginia and northeastern North Carolina, northern South Carolina and West Texas, and they are accountable for ~ 15% of total US production. Virginia-type also gained popularity in other world regions, such as the Near East (mainly Egypt and Israel), grown for in-shell production and export to Europe. The diverse environmental conditions across peanut growing regions resulted in the selection of peanut cultivars with varied TTM. In areas with limited water supply or end-of-season cool temperatures and early frost^9^, the peanut maturation process is retarded which often results in incomplete pod filling, low yield, grade and quality (including low oleic to linoleic acid ratios). In these regions, early maturing peanut varieties are preferred^10,11^. Therefore, most of the Virginia peanuts were historically developed as early-maturing (130–140 DPP) or medium-maturing (140–150 DPP), while the Runners are mostly late-maturing (150–170 DPP) (https://issuu.com/onegrower/docs/peanut_grower_2019_variety_guide)^12^.

TTM is an important breeding objective, particularly for introducing early maturation to both Virginia- and Runner-type idiotypes, with better adaptation to specific geographical regions, yield characteristics and agronomic performance. However, introducing early maturation to late-maturing cultivars is a challenging task due to the insufficient knowledge regarding the genetic and molecular mechanisms that control TTM. Peanut maturity level was reported as a quantitative trait with low heritability^13,14^ and influenced by many genes and environmental factors^15,16^. Domesticated peanut has a narrow genetic base, resulting from the bottleneck of a single hybridization event that gave rise to this species and the crossing barriers between tetraploid cultivated peanut and diploid wild ancestors due to ploidy differences^17^. Apart from the low polymorphism among cultivars, another hurdle is phenotyping TTM due to the unique underground formation of peanut pods. The hull-scrape method^18^ is commonly used to determine TTM in peanut, but this method is laborious and may be subjective.

Currently, only a few attempts to define genomic loci that control TTM were made using breeding materials from ssp. *fastigiata* × ssp. *hypogaea* crosses^19–21^. Most of these were with low-density genetic maps and resulted in identification of relatively small effect QTLs for early maturation. Through the advancement in SNP array technology^22,23^, a dramatic increase in number of genetic markers alleviated the existing limitation in constructing high-density genetic maps. A recent study involving Virginia-late × Virginia-early background used a SNP-based mapping approach to identify significant QTLs for TTM, some of which were associated with harvest index, pod yield, and branching habit trait related QTLs^7^.

In this study, a SNP-based linkage map was constructed for an F_7:9_ RIL population derived from Runner (late) × Virginia (early) market-type background. A QTL analysis was performed for TTM with a two-year field experiment phenotyping data set. New QTLs associated with TTM are reported, and the translation of these QTLs into user-friendly marker platforms for facilitating marker-assisted selection is suggested.

## Results

### Phenotyping of the parents and the RIL population for TTM

**Figure 1:**
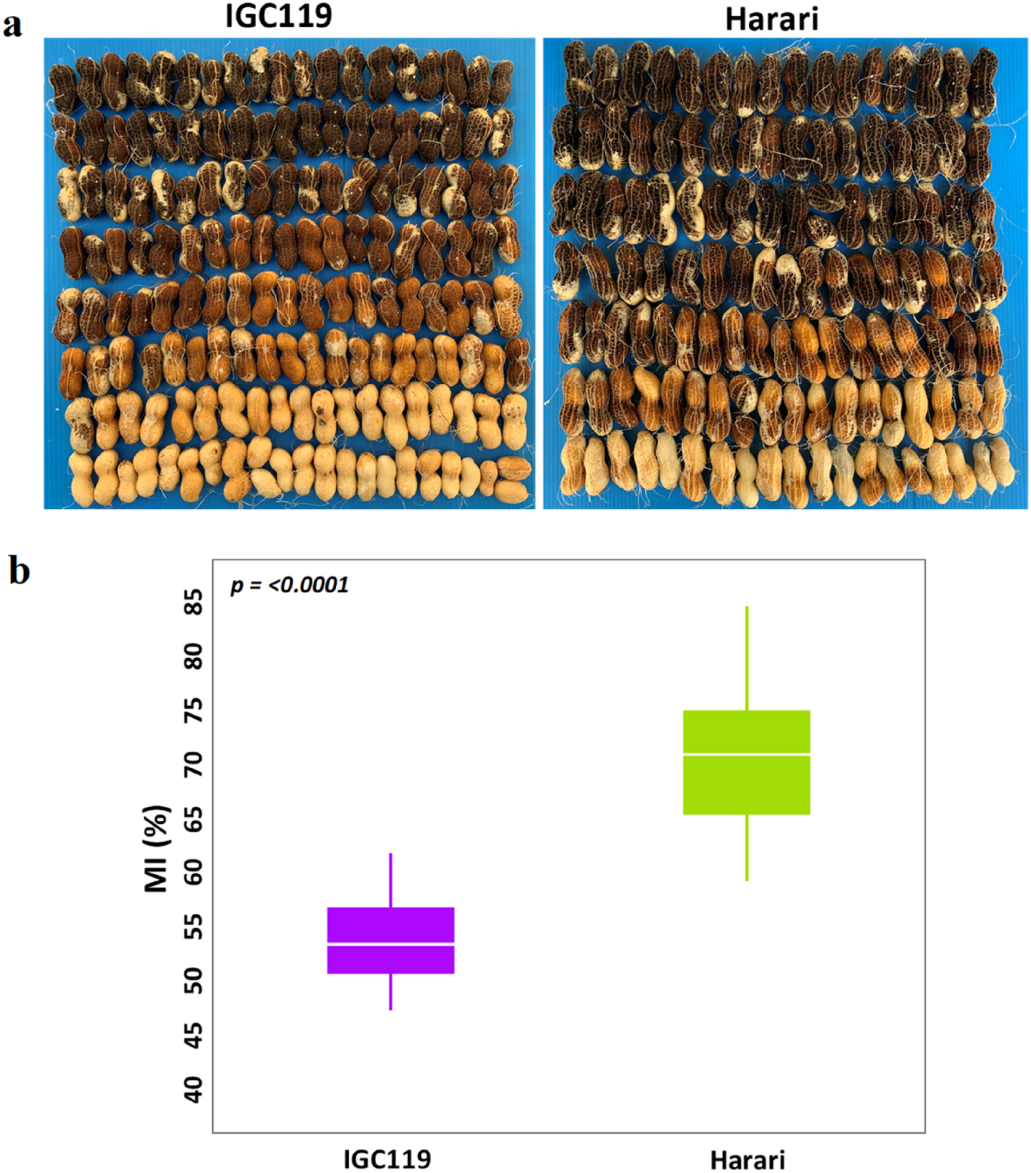
Phenotypic characterization of MI trait in ‘IGC119' and ‘Harari’. (**a**) Image of mesocarp color from pressure washed fresh pods harvested from ‘IGC119' and ‘Harari’. (**b**) Comparison of MI between ‘IGC119’ and ‘Harari’. Data are shown as a boxplot from ten replicates of each genotype. The Student’s t-test was used to determine the *P* value. MI, maturity index (%).

A RIL population that was developed from a cross between IGC119 (late-maturing) and Harari (early-maturing) cultivars (Fig. 1a) was used for the investigation. TTM was indicated as the maturity index (MI), determined by the percentage of pods with black and brown mesocarp. Data were collected from field experiments in two seasons (Supplementary Dataset: Table S1). A highly significant difference was found between the parental lines in MI (*P* ≤ 0.0001), with 53.9 ± 1.97 and 70.7 ± 1.97 for IGC119 and Harari, respectively (Fig. 1b).

**Figure 2:**
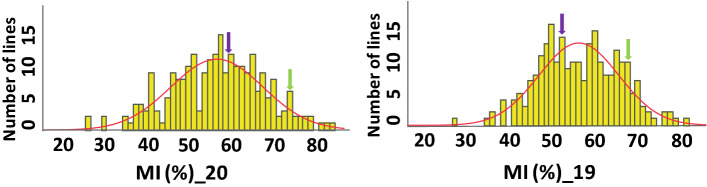
Phenotypic distribution of MI trait in 2019 (left panel) and 2020 (right panel). The Y-axis corresponds to the number of RIL lines in each range of MI, and X-axis corresponds to the MI value based on the average of three replicates. Arrows indicate the phenotypic values for 'IGC119' (purple) and 'Harari' (green). A normal distribution curve is indicated by the red line.

The MI trait was distributed normally or close to normal in the RIL population (Fig. 2; Table 1). Parental values of the MI trait were within the range of the RILs. Transgressive segregation of MI was observed among the RIL population in both years (Fig. 2). ANOVA analysis showed significant effect on MI from the blocks, RIL, and RIL × year interaction but not from year (Table 2). Due to the significant effect from RIL × Year interaction, QTL analysis was performed separately with data from each year. The broad-sense heritability estimate for MI was 0.44, indicating a moderate but significant genetic component underlying this trait.

**Table 1:** Summary statistics of MI trait among parents and RILs.

	Variables	Parents			RILs			
		IGC119	Harari	Student t-test	Mean ± SD	Minimum	Maximum	Sig. of A–D test ^b^
2019	MI (%)^a^	51.3	67.3	0.0004*	55.8 ± 9.3	26.4	80.6	0.109
2020	MI (%)	58.2	72.6	0.0078*	56.1 ± 10.9	25.2	80.02	0.779

^a^MI (%), maturity index; ^b^significance for normality test by Anderson—darling test; *significant at *P* < 0.01.

**Table 2:** Analysis of variance and heritability for MI trait for the IGC119 × Harari RIL population across 2 years.

Trait	Variables	DF	Mean square	F ratio	P-value	H^2i^
MI^a^	Block (year)	4	6805.27	86.09	< 0.0001*	0.44
	Year	1	27.56	0.34	0.555^ ns^	
	RIL	242	506.72	6.41	< 0.0001*	
	RIL × year	242	105.29	1.33	0.001*	
	Error	967	79.04			

Block [year] indicates the nested effect of the Blocks within each year.

^a^*MI* maturity index, ^i^Broad sense heritability, ^*^significant at *P* < 0.01; ^ns^non-significant.

### Construction of a genetic map

Genotyping of parents IGC119, Harari and RIL lines was performed with version 2 of the Axiom *Arachis*_SNP array consisting of 47 K SNP markers (Thermofisher Scientific). After initial filtering, a set of 3116 polymorphic SNP markers were obtained between the two parental lines. After filtering for 65–35% call rates in the RIL population and removing the duplicates, 2744 SNPs were retained. Subsequently, a genetic map was constructed with 243 RILs and 2744 SNPs as input (Supplementary Dataset: Table S2). Following the JoinMap software parameters, 768 loci were excluded for exceeding the chi-square test threshold, and 551 loci were excluded due to loci-similarity. Therefore, the genetic map constructed contained 1425 markers distributed on 24 linkage groups (LG), covering a total of 950.2 cM (Table 3; Fig. S1). The number of LG was larger than 20 (the chromosome number), a common situation in genetic mapping in peanut^7,24,25^ due to low polymorphism and the complicated tetraploid genome.

**Table 3:** Description of genetic linkage groups. Physical distance was determined by BLAST alignment of markers against the *A. hypogaea* reference genome (version 2) (peanutbase.org).

Linkage group	Chromosome assignment	No. of SNPs	Map distance (cM)	Average loci interval (cM)	Physical length (Mbp)	Average physical interval (Mbp)	Total length *A. hypogaea* genome (Mbp)	Coverage ratio	Recombination rate (cM/Mbp)
A01	Arahy.01	37	64.4	1.7	109.1	2.9	112.42	0.97	0.57
A02	Arahy.02	17	27.7	1.6	3.8	0.2	102.98	0.04	0.27
A03_1	Arahy.03	15	20.6	1.4	4.2	0.3	143.81	0.03	0.14
A03_2		7	49.6	7.1	117.8	16.8	143.81	0.82	0.34
A04	Arahy.04	65	92	1.4	126.64	1.9	128.8	0.98	0.71
A05_1	Arahy.05	108	13.1	0.1	75.3	0.7	115.93	0.65	0.11
A05_2		11	24.9	2.3	5.1	0.5	115.93	0.04	0.21
A06	Arahy.06	239	49.2	0.2	106.4	0.4	115.5	0.92	0.43
A07	Arahy.07	246	75.9	0.3	77.2	0.3	81.12	0.95	0.94
A08_1	Arahy.08	19	31.6	1.7	13.6	0.7	51.9	0.26	0.61
A08_2		17	21.2	1.2	4.1	0.2	51.9	0.08	0.41
A09	Arahy.09	15	63.2	4.2	104.6	7.0	120.52	0.87	0.52
A10	Arahy.10	69	50.8	0.7	109.9	1.6	117.09	0.94	0.43
B01_1	Arahy.11	54	48	0.9	141.2	2.6	143.9	0.98	0.33
B01_2		6	15.9	2.7	1.7	0.3	143.9	0.01	0.11
B02	Arahy.12	30	19	0.6	97.6	3.3	120.58	0.81	0.16
B03	Arahy.13	28	107.3	3.8	142.9	5.1	146.73	0.97	0.73
B04	Arahy.14	36	39.5	1.1	122.1	3.4	143.24	0.85	0.28
B05	Arahy.15	8	45.1	5.6	141.6	17.7	160.88	0.88	0.28
B06	Arahy.16	185	48.5	0.3	139.2	0.8	154.81	0.90	0.31
B07	Arahy.17	3	14.1	4.7	1.3	0.4	134.92	0.01	0.10
B08	Arahy.18	35	17.6	0.5	115.9	3.3	135.15	0.86	0.13
B09	Arahy.19	6	1.3	0.2	17.6	2.9	158.63	0.11	0.01
B10	Arahy.20	169	9.7	0.1	105.9	0.6	143.98	0.74	0.07
	Mean	59.375	39.6	1.9	78.5	3.1	124.5	0.6	0.3
	Total	1425	950.2	44.5	1884.7	74.1	2988.4	14.7	8.2

The 24 LGs ranged in size from 1.3 cM (B09) to 107.3 cM (B03). The average number of markers per LG ranged from 59, reaching 246 loci in LG A07. The average distance between neighboring loci was 1.9 cM, ranging from 0.1 cM in LGs A05_1 and B10 to 7.1 cM in A03_2 (Table 3). Aligning the 1425 mapped markers to the *A. hypogaea* pseudomolecules (peanutbase.org) resulted in a total physical distance of 1884.7 Mbp and an average physical interval between markers of 3.1 Mbp (Table 3; Fig. S1). The percentage coverage by the pseudomolecule by each LG varied; eight groups covered more than 90%, six groups more than 80% and two more than 60% of a pseudomolecule. The average recombination rate was found to be 0.3 cM/Mbp. Linkage group A07 had the maximum recombination rate, while the LGs B09, B10, B07, B01_2 and A05_2 had the lowest recombination rates. The genetic map quality was evaluated by analyzing the loci collinearity to their physical positions (Mbp) in the *A. hypogaea* genome. As expected, the saturation of the markers in the distal regions was higher than in the pericentromeric ones and low to moderate recombination was observed in the center of LGs (Fig. S1).

### QTL identification

**Figure 3:**
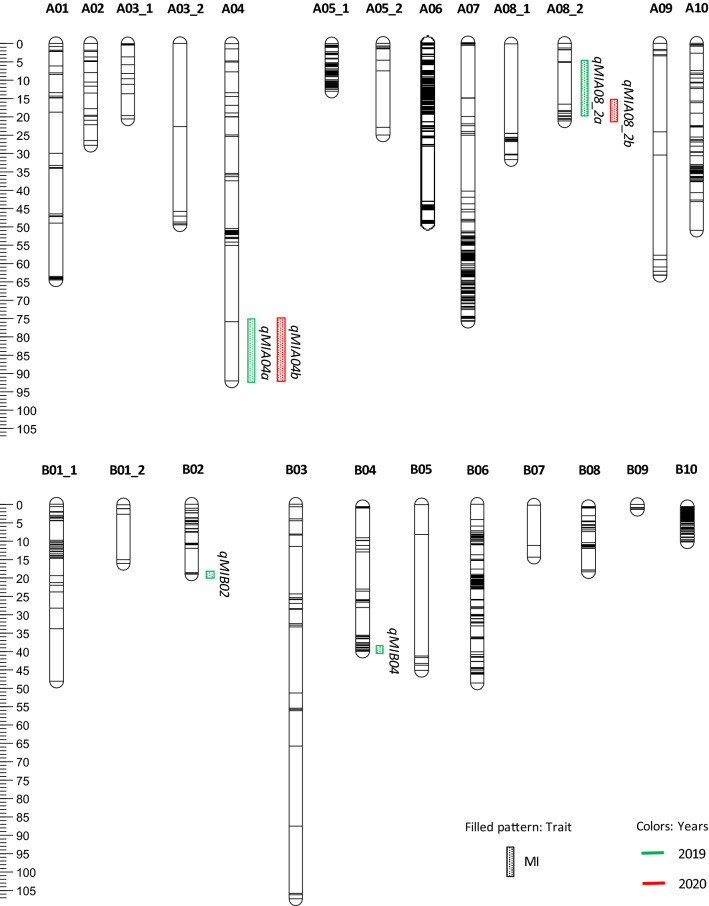
An overview of the genetic map and the QTLs identified for MI trait in 2 years. MI, maturity index.

Mapping the MI trait resulted in six QTLs, four QTLs in 2019 and two in 2020, respectively, with the LOD scores ranging from 3.74 to 6.44, explaining 6.8 to 11.5% of the phenotypic variance (PVE) (Fig. 3; Table 4). Two consistent QTL regions were found in both years. One (*qMIA4a* and *qMIA4b*) was observed on LG A04 between AX-176819644_A04–AX-147221341_A04, spanning 7.9 Mbp, with PVE values of 11.5 and 8.1% for 2019 and 2020, respectively (Fig. 3; Table 4). The other consistent QTL (*qMIA08_2a* and *qMIA08_2b*) region was observed on LG A08_2 within marker interval of AX-177639781_A08–AX-176821672_A08, spanning 1 Mbp, explaining 7.3 and 8.2% PVE in 2019 and 2020, respectively. The other two QTLs were identified on LG B02 (*qMIB02*) and LG B04 (*qMIB04*), which were significant only in 2019 (Fig. 3; Table 4). Alleles from the early-maturing parent, Harari, contribute to the high percentage of mature pods measured by MI for all six QTLs, *qMIA04a* and *qMIA04b*, *qMIA08_2a* and *qMIA08_2b*, *qMIB02* and *qMIB04* (Table 4).

**Table 4:** QTLs identified for MI trait in the Harari × IGC119 RIL population.

Trait	Environment	QTL	LG^a^	Flanking markers	LOD	Additive effect*	PVE^b^ (R^2^)	Position range (cM)	Physical position (Mbp)
MI	2019	*qMIA04a*	A04	AX-176819644_A04–AX-147221341_A04	6.44	3.20702	11.5	75.75–92.00	118.68–126.64
MI	2019	*qMIA08_2a*	A08_2	AX-177637914_A08–AX-176821868_A08	4.02	2.53183	7.3	5.19–20.84	48.42–51.44
MI	2019	*qMIB02*	B02	AX-176794798_B02–AX-176812478_B02	4.96	2.79835	9	18.63–19.02	105.81–105.9
MI	2019	*qMIB04*	B04	AX-176802465_B04–AX-176799466_B04	3.74	2.43951	6.8	39.06–39.46	56.44–57.01
MI	2020	*qMIA04b*	A04	AX-176819644_A04–AX-147221341_A04	4.48	3.15765	8.1	75.75–92.00	118.68–126.64
MI	2020	*qMIA08_2b*	A08_2	AX-177639781_A08–AX-176821672_A08	4.53	3.13725	8.2	16.63–21.24	50.29–51.29

^a^*LG* linkage group, ^b^*PVE* Phenotypic variance explained; *Additive effect for all the QTLs contributed by parent 'Harari'. MI, maturity index.

The gene models within the two consistent QTL regions on chromosomes A04 and A08 were extracted from the PeanutBase Tifrunner version 2 reference genome (https://mines.legumeinfo.org/peanutmine/template.do?name=region_genes&scope=global). The first QTL region (defined as the region I) on chromosome A04, with flanking markers AX-176819644_A04–AX-147221341_A04, spanned a genomic distance of 7.9 Mb (physical positions 118680323–126646480) and enclosed 537 gene models (Supplementary Dataset: Table S3). The other QTL region (defined as the region II) on chromosome A08, with flanking markers AX-177639781_A08–AX-176821672_A08 covered a genomic distance of 1 Mb (physical positions 50299697–51293132) with 90 gene models (Supplementary Dataset: Table S4). A total of 15 SNPs were detected in these two QTL regions (4 SNPs in region I and 11 SNPs in region II); none of the SNP changes led to alterations in protein-coding sequences (Supplementary Dataset: Table S5).

## Discussion

Time to maturation is a significant factor that directly affects the growing duration and determines the final yield and quality in many crops. Usually, late maturation is associated with increased crop yield and prolonged pod-filling processes^26,27^, whereas early maturity is associated with better adaptation to regions where seasons are constricted by drastic changes in environmental conditions^28^. In legumes, two major developmental aspects are considered to control TTM—the flowering time and inflorescence determination^29–34^. Peanut exhibits a unique genetic system for TTM among legumes. Although peanut is considered a short-day plant^35^, earlier studies clearly show that flowering is minimally affected by the photoperiod that has a very small effect on TTM^35^. While variation in flowering patterns between ssp. *fastigiata* and *hypogaea* exists, domesticated peanut exhibits indeterminate lateral shoot tips^36^; therefore, the "classic" model of inflorescence architecture contributing to TTM cannot be applied to domesticated peanut.

Despite its agronomic importance and unique genetic system, scientific advances in peanut maturity genetics is still in its infancy. Identifying genome-wide markers and polymorphism among populations is essential to study quantitative and complex phenotypic traits^37^. Indeed, QTL mapping is a powerful strategy to elucidate the novel and stable loci for molecular breeding application through molecular marker-assisted selection in peanut. However, low polymorphism in domesticated peanut has hampered the discovery of novel QTL associated with target traits. This situation has greatly improved in recent years, with the availability of the diploid^2^ and the domesticated tetraploid^20,38^ reference genomes and the discovery of new markers. SNPs represent one such marker group, which constitutes an abundant source of genetic variation at the genome level, significantly improving genome coverage and marker saturation^39^. So far, much of the effort on peanut SNP-based genotyping and trait mapping was made on several agronomically important traits such as pod and seed traits^40–42^ and disease-related traits^43–48^. In this study, with the application of SNP microarray technology and focused phenotyping, two stable QTL for TTM was exposed in a recombinant inbred population from a Runner × Virginia cross with the parental TTM differing by ~ 10–15 days.

Phenotypic distributions of the MI trait among the RIL population extended beyond parental values suggesting the presence of transgressive segregation. The broad-sense heritability for MI was found to be moderate (~ 0.44), similar to our recent publication^7^ and somewhat higher than other studies^16,17^. Six QTL were identified on chromosomes A04, A08, B02, and B04 in two environments. Four QTLs were detected in 2019 and 2 QTLs in 2020. We could not find any phenotypic correlation or common QTLs with other traits like branching habit, pod size and the average number of pods per plant (data not shown), suggesting no association with MI trait in this population. In particular, pod size, a strong candidate component trait that can affect MI in peanut (smaller pods usually mature earlier), was even the opposite in the two parental lines. Harari, which has a bigger pod size, is earlier matured than IGC99, which has a smaller pod size (Fig. 1). Also, based on the recent finding regarding the association between MI and harvest index (HI) in Virginia-type peanut^7^, we phenotyped HI in 2019, but did not find any association between MI vs. HI traits (so HI was excluded in 2020 because of its laborious data collection). This also contradicts earlier reports in other legume crops^49–51^, showing a strong correlation between HI and maturity due to the a higher sink/source level in early maturing genotypes.

The identified QTLs have small to moderate effects on MI (Table 4), and two of them *qMIA04a,b and qMIA08_2a,b* were consistently detected in both years. Most of the QTLs reported in the study are novel and different from those reported for TTM by others^7–10^. However, one consistent QTL from the current study, *qMIA04a,b* (flanking markers: AX-176819644_A04–AX-147221341_A04, physical position: 118680323–126646480), overlapped with the previously reported consistent QTLs *qMIA04a* (flanking markers: AX-176802283_A04–AX-176815499_A04, physical position: 117632573–125599371), and *qMIA04b* (flanking markers: AX-176819644_A04–AX-176815499_A04, physical position: 118680323–125599371) in our Virginia × Virginia background^7^. This overlapping QTL has the early maturing Harari as the common parent in both RIL populations, signifying the importance of this stable QTL region for Harari-based MAS breeding in different late-maturing backgrounds. Yet, it is important to note that this QTL region is still specific to a single genetic background. Harari has some *fastigiata* related heritage in its genetic background^7^, indicating that this QTL can be anticipated to be consistent across varying genetic backgrounds. However, further work is needed to inspect this contribution in other early-maturing Virginia-type germplasms.

The inadequate marker density and the low polymorphism existing among both parents used in the study limited our ability to predict target candidate genes for TTM. Also, a general enriched GO annotation study did not show any significant biological processes that are over-represented in these loci (data is not shown). Therefore, more efforts should be made to find more SNPs across this region to identify significant candidate genes for TTM. Since the cost of sequencing dropped dramatically, it may be feasible to significantly enrich marker density by sequencing the whole genome^52^. The QTLs for MI that were detected in only one year and showed no association with other traits may be largely affected by environmental factors, and further validation will be required from multi-location experiments and development of additional populations using distantly related crossing parents. The information and materials generated here, particularly on the stable cross-population QTL, can be integrated into informed targeting of peanut idiotypes by indirect marker-assisted selection.

## Materials and methods

### Plant material

The RIL population used in the study was developed from a cross between a Runner-type cv. IGC119 and the Virginia-type cv. Harari, differing in TTM by 10–15 days. RILs were developed by a single seed descent (SSD) method, up to F_7_, and multiplied as bulks for another two generations (F_7:9_). IGC119 is part of the Israeli Groundnut Collection, and it is the local name for the runner cv. GK-7 HIGH OLEIC (PI 599592). It is a late-maturing cultivar with small, smooth, hard pods and high-oleic kernels. Harari is an early-maturing cultivar grown in northern Israel. Harari has big pods with reticulated and soft shells targeted for the local shelled peanut industry. Its growing season is limited by the regional conditions of late sowing time (due to the double-cropping system) and early harvest (due to autumn rainfalls). IGC119 has a spreading growth habit, while Harari is a bunch type. Common traits between IGC119 and Harari include flowering pattern, lateral branch length and flowering rate.

Field experiments were performed with 243 F_7:9_ RILs in two successive years. The first year was planted in April 2019 in the Magen, Western Negev, Israel (33°11′17.7″N 35°34′25.6″E), and the second in April 2020 in Urim, Western Negev, Israel (31°20′27.4″N 34°29′46.1″E). Both locations are characterized by fine sandy-loam soil. A randomized complete block design with three replications was applied in the field experiments in both years. Plot size of 2 m × 1 m consisted of two rows on a bed, rows spaced 90 cm apart and seeding rates of 10 seeds/m^2^ (total of 20 plants/plot) was implemented. Parental lines were grown as control plots in six replications. Field experiments were maintained under full irrigation, and all recommended agronomic practices were carried out as previously described^53^. All rights for the plant material, including the parental and the RILs, belong to the Hovav laboratory and are part of the ARO breeding program.

### Phenotyping the MI trait and statistical analysis

MI was evaluated at ~ 145–150 days post-planting (DPP). The precise evaluation date was determined as described^7^, by sampling both parental lines from 130 DPP every few days until Harari reached ~ 60% maturity on average. This approach was preferred to capture the widest variation in TTM among the RILs. The hull-scrape method^21^ was applied to measure the maturity level as described^7^. Briefly, 2–3 random plants per plot were sampled, and the exocarp was removed from the pods using a PICO water pressure machine (Idromatic®, Italy) (Fig. 1a). Pods then were separated into five categories based on mesocarp color: white, yellow, orange, brown, or black. The maturity index (MI) was calculated as the percentage of pods in the brown and black categories. In total, 729 MI measurements were taken each year.

The normality of distribution of MI among the RILs was tested by Anderson–Darling test. The Student's t-test was used to determine statistical differences between the parental lines. ANOVA model was used in the same manner as described^7^ to define the effects of RIL, Year, Year X RIL and Block. To calculate the broad-sense heritability, all effects in the linear model were defined as random, and heritability (*H*^2^) was estimated with the equation *H*^2^ = σ_g_^2^/(σ_g_^2^ + σ_ge_^2^ + σ_e_^2^), by the ANOVA analysis with QTL IciMapping v4.2 (http://www.isbreeding.net/software/?type=detail&id=29) as described^40^. The σ_g_^2^, σ_e_^2^ and σ_ge_^2^ denoted the variances of genotypes (G), environment (E) and interaction of genotypes and environments (G × E). Distribution, histograms, and boxplots were performed with JMP® Pro 15 (SAS Institute Inc., Cary, NC, 1989–2019).

### Genotyping, SNP filtering and genetic map construction

Genomic DNA was extracted from young leaflets from each RIL and the two parents using GenElute™ Plant Genomic DNA Miniprep Kit (Sigma-Aldrich, USA). DNA was quantified with Qubit (Invitrogen; CA, USA). DNA samples were diluted according to protocol guidelines to 40 ng/μL and genotyped through Affymetrix Axiom_*Arachis*2 SNP array comprising 47,837 SNPs, separated into their AA and BB subgenomic origin^23,54^. Genotyped data was analyzed by the Axiom analysis suite Software 3.1^25^. Call-rate frequencies of 65–35% were applied in retaining polymorphic homozygous (AA and BB) and polymorphic heterozygous (AA or BB and AB) SNPs among the RILs resulting in retention of 2744 SNPs for further analyses.

The genetic map was constructed with 243 RILs and 2744 SNPs as input. The linkage map (LG) was constructed using Joinmap v4.1^55^ with regression algorithm to calculate locus genotype frequency, retaining only SNPs with a chi-square *P*-value ≤ 0.05 (1 degree of freedom). Groupings were established according to an independent LOD that started at 2.0 and ended at 10. Map distances were estimated using Kosambi mapping function. The graphical representation of the LG was obtained through MapChart v2.3^56^. Confirmation of loci positions was made as previously described^39^ with few modifications (BLASTN (e-value < 1 × 10^− 18^) and mismatch of less than 2). LGs developed were assigned to the pseudo-molecules of the tetraploid *A. hypogaea* cv. Tifrunner^36^ (https://peanutbase.org) (Table 3). LG where more than 51% of the representative SNPs localized to a particular chromosome were assigned to that chromosome. Assessment of the quality of the genetic map was performed with a collinearity analysis using the genetic distances (cM) versus the physical positions (Mbp).

### QTL mapping

MI trait mapping was performed with the mean phenotypic data collected each year on 243 RILs using MapQTL v6^57^ (Supplementary Dataset: Table S1). Interval mapping used a regression algorithm with significance thresholds calculated by a permutation test of α < 0.05 and n = 1000. The MI trait QTLs with LOD score of > 3 were manually assigned to the LG. The QTL nomenclature is as follows "q" as QTL, followed by MI trait, the last digit represents the LG, and repetition of the QTL in alphabetical order if in both years. Physical positions from the *A. hypogaea* genome were obtained from markers flanking the QTL.

### Permission statement

All the experiments on plants, including the collection of peanut seed materials, were performed in accordance with relevant guidelines and regulations.

## Supplementary Information

Supplementary Figures.

Supplementary Tables.

## Data Availability

All data generated or analysed during this study are included in this published article [and its supplementary information files].
