# Supplementary material for: A first insight into the genetics of maturity trait in Runner × Virginia types peanut background
Source: Sci Rep. 2022 Sep 10;12:15267. doi: 10.1038/s41598-022-19653-z (PMC9464196; doi:10.1038/s41598-022-19653-z)
Supplement: Supplementary file 1 — Supplementary Figures. [file 41598_2022_19653_MOESM1_ESM.docx]

**A first insight into the genetics of maturity trait in Runner X Virginia types peanut background**

Srinivas Kunta^1,2^, Pragna Parimi^2^, Yael Levy^1^, Chandrasekhar Kottakota^1^, Ilan Chedvat^1^, Ye Chu^3^, Peggy Ozias-Akins^3^, Ran Hovav^1^

^1^ Department of Field Crops, Institute of Plant Sciences, Agriculture Research Organization-The Volcani Center, HaMaccabim Road, POB 15159, Rishon LeZiyyon 7505101, Israel

^2^ Faculty of Agricultural, Food and the Environmental Quality Sciences, The Hebrew University of Jerusalem, POB 12, Rehovot 7610001, Israel

^3^ Department of Horticulture and Institute of Plant Breeding, Genetics and Genomics, University of Georgia, Tifton, GA 31793, USA

**Figure S1.** The x-axis measures the genetic distance (cM) of markers on each linkage group (LG). The y-axis represents the genome position (Mbp) of genetic markers based on Tifrunner reference genome. Black dots represents the markers of respective chromosome with the LG, red dots to the homeologous chromosome and black circles represents the markers from other chromosome.
